# Research on a Measurement Method for Downhole Drill String Eccentricity Based on a Multi-Sensor Layout

**DOI:** 10.3390/s21041258

**Published:** 2021-02-10

**Authors:** Hongqiang Li, Ruihe Wang

**Affiliations:** 1 School of Petroleum Engineering, China University of Petroleum, Qingdao 266580, China; b13020045@s.upc.edu.cn; 2 Sinopec Shengli Drilling Technology Research Institute, Dongying 257000, China

**Keywords:** drill string eccentricity, drill string dynamics, ultrasonic caliper while drilling, fiber-optic gyro, logging while drilling, imaging measurement, multi-sensor combination

## Abstract

The drill string used in drilling is in a complex motion state downhole for several kilometers. The operating attitude and eccentricity of the downhole drill string play important roles in avoiding downhole risks and correcting the output of the imaging measurement sensor while drilling (IMWD). This paper proposes a method for measuring eccentricity while drilling using two sets of caliper sensors coupled with a fiber-optic gyroscope for continuous attitude measurement, which is used to solve the problem of the quantitative measurement of complex eccentricity that changes in real-time downhole. According to the measurement and calculation methods involved in this article, we performed simulations of the attitude of the drill string near where the IMWD tool is located in the wellbore under a variety of complex downhole conditions, such as centering, eccentricity, tilt, buckling, rotation, revolution, etc. The simulation and field test results prove that the distance between the imaging while drilling sensor and the borehole wall is greatly affected by the downhole attitude and revolution. The multi-sensor layout measurement scheme and the data processing based on the above-mentioned measurement involved can push the drill collar movement and eccentricity matrix specifically studied downhole from only qualitative estimation to real-time measurement and quantitative calculation. The above measurement and data processing methods can accurately measure and identify the local operating posture of the drill string where the IMWD sensor is located, and quantitatively give the eccentric distance matrix from the measuring point to the borehole wall required for environmental correction of the IMWD sensor.

## 1. Introduction

With the development of petroleum energy, imaging while drilling has become an important technology to improve the penetration rate of oil and gas reservoirs, accelerate drilling speed, and reduce drilling cost. During drilling, imaging measurement sensors while drilling (IMWD) mounted in special drill collars continuously scan the borehole wall and the formation behind the borehole wall several kilometers downhole to obtain changes in the wellbore surface and geological information on the formation behind the borehole wall, such as natural gamma ray, neutrons, density, and resistivity. This information is used to reveal the geology of the drilling and to analyze and find recoverable reservoirs. In the measurement project, the signal of the sensor needs to pass through the annulus between the drill collar and the borehole wall before reaching the borehole wall. Because the diameter of the drill string is smaller than the diameter of the wellbore, the drill string is in a complex operating state downhole and is under the conditions of gravity; the additional reaction force of the bit in contact with the wellbore wall; the friction resistance caused by the drill string in contact with the wellbore wall; and other composite factors, such as eccentric self-rotation, revolution around the shaft wall, sinusoidal bending, vortex, repeated vibration, and other operating states. At the same time, because the borehole wall will expand, shrink, and collapse in the process of drilling [1,2], resulting in uneven borehole wall, etc., it is more difficult to capture the downhole running track of the drill collar in real time and obtain the eccentric position of the sensor [3,4,5,6].

Therefore, the distance between the IMWD sensor and the borehole wall is also changing constantly, and it is difficult to obtain the distance change to provide a compensation basis for the eccentricity correction of the IMWD sensor. Drill string dynamic characteristics have been studied for decades, and very important research results have been obtained from qualitative measurements, providing valuable parameters for reducing downhole drill string failure and optimizing the drill string structure [7,8,9,10,11,12]. However, the previous studies on this topic were all macroscopic. The posture of the drill string constantly changes over time due to the various stress conditions of the bottom hole assembly (BHA). For the measurement of gamma imaging, neutron density, and electromagnetic resistivity, the eccentricity distance has a great influence on the measurement results [13,14,15,16,17,18,19]. In order to obtain the real formation information accurately, modified maps of the sensors with different eccentricity distances are given. The traditional wireline logging uses the multi-arm diameter to obtain the distance from the instrument center to the borehole wall. It is very difficult to measure the eccentric distance of the drill collar in the eccentric and complex rotation of the drill collar.

In order to identify the running state of the drill string in real time, the eccentric distance of the drill string where the imaging sensor is located is given. A measurement method that can obtain the eccentric distance of the imaging sensor at a high frequency is needed. The multi-sensor layout proposed in this paper is mainly used to measure the real-time eccentricity state of the IMWD sensor and provides the real-time eccentricity distance for sensor eccentricity compensation. The downhole drill string eccentricity measurement method designed in this paper uses two sets of ultrasonic diameter sensors installed at both ends of the special drill collars for imaging measurement sensors while drilling and a middle center attitude sensor to measure and obtain the motion characteristics and eccentricity state of the drill string in real time.

The main components of this paper can be divided into the following parts: (1) In the second section, the composition of the drill string during the directional measurement near the bit end while drilling is introduced, and the factors leading to eccentricity are expounded. Then, a method for the calculation of the drill string running characteristic and the possible eccentric motion characteristic is introduced. (2) In the third section, the measuring principle of the multi-sensor layout method for measuring the drill string eccentricity state proposed in this paper is introduced, and methods for the calculation of the key parameters, such as drill string eccentricity angle, revolution velocity, and rotation velocity, are given. The methods for the calculation of each orientation eccentricity matrix at different measuring points of the drill collars for imaging while drilling are further obtained. (3) In the fourth section, according to the multi-sensor layout measurement principle, the above-mentioned eccentric running state is simulated, and the distance of the borehole wall from the measuring point has been measured for each combination of depth and angle. The use of the above method and experiment prove that, despite the complicated operation of the downhole drilling string, the above method can capture the real-time running characteristics of the drill string, can provide the real-time measurement of the eccentric distance while drilling, can image the raw data for real-time correction while drilling, and can accurately identify the underground geological characteristics to provide technical support.

## 2. The Composition and Operation Characteristics of the Downhole Drill String

### 2.1. Composition of the Bottom Drill String

**Figure 1:**
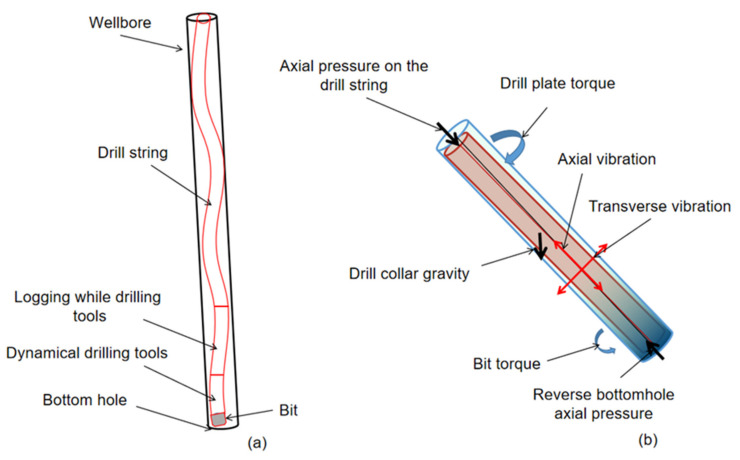
(**a**) Position and force of drill collars while drilling in the wellbore; (**b**) Schematic diagram of drill collar force.

Figure 1a shows the typical BHA for logging while drilling (LWD) and directional drilling under normal conditions. The BHA consists of the bit, the downhole motor (DM) with a bend, the IMWD drill collar, and the drill pipe. As can be seen from Figure 1, due to the gap between the wellbore and the drill string, the drill string is in a state of tilt and bend in the wellbore under complex stress conditions. These tilt and bend states also vary due to changes in weight on bit, torque, and drillability. Specifically, we focused on the movement characteristics and the eccentric displacement of the drill collar (IMWDDC) in the wellbore, as shown in Figure 1b. The drill collars in this part are closer to the drill bit, and different diameters of the drill collars in which the instrument is located will cause different degrees of bending under pressure. In order to obtain the eccentric distance between the imaging measuring sensor on the drill collar and the borehole wall, it is used to correct the influence of the downhole measuring environment on the sensor. It is necessary to measure, calculate, and analyze the attitude of IMWDDC in the wellbore and the distance of each sensor to the wellbore wall in real time.

The commonly used BHA and bit sizes for measurement while drilling are shown in the Table 1, including the drill bit, DM, and drill collar of the instrument while drilling (DCWD). The table gives the possible magnification of the hole (MH), the range of distance from the sensor to the wellbore wall (RDSW), and the degree to which the drill collars bend under stress.

It can be seen that there is a large gap between the wellbore and the drill string due to the difference between the drill bit diameter and the drill collar diameter of the instrument while drilling. This is also due to the drilling tool mud fluid and carrying the broken cuttings of the channel. The mud performance and type and thickness of the channel have a great impact on the original output of the imaging while drilling measuring instrument. In particular, the distance between the wellbore wall and the drill string increased due to the expansion of the wellbore.

### 2.2. Causes of Drill String Eccentricity

As shown in Figure 1a,b, the drill string is subjected to complex forces such as its own weight, the reaction force of the bit in contact with the formation, and the torque of the drill string and the bit in contact with the formation. The drill string rotates under the combined action of various forces, which drives the drill bit to contact the cutting strata at the bottom of the hole to break rock. Similarly, the drill string is bent and eccentric under these combined forces. American Woods and Lubinski studied and obtained the critical equation for the spiral bending caused by the dead weight of the drill string [20]. In the 1980s, Dellinger regressed the Lubinski test curve and reanalyzed the calculation equation [21]. In 1984, Dowson first proposed the load equation of the sinusoidal bending of a drill string [22]. On this basis, Yu Yongnan and Huang Tao et al. from the University of Petroleum (East China) continued their research and experiments and obtained the critical calculation equation for sinusoidal buckling at the University of Petroleum [23], as shown in Equation (1).

(1) $$F_{hel}=2.4175\sqrt{\frac{EIq_{m}\sin Inc}{r_{DD}}}$$

where *q_m_* is the floating weight of the drill string in mud, N/m; *E* is the elastic modulus of steel, Pa; *I* is the moment of inertia of the drill collar section axis; *r_DD_* = 0.5 (DH-DC) is the apparent radius, where DH is the hole diameter and DC is the drill string diameter; Inc is well inclination.

Through the above research, we can roughly obtain the downhole bending state of the drill string and some contact points of the borehole wall. After learning the above information, the BHA, the weight on the bit, and the rotary speed can be adjusted to prevent the instability of the drill string and avoid downhole risks. It is impossible to use the above theoretical analysis to calculate the real-time operating attitude of the DCWD in the wellbore, as shown in Figure 1b, and to obtain the eccentricity state and the precise distance from each measurement point above to the wellbore wall.

### 2.3. Operational Characteristics of the Drill String

**Figure 2:**
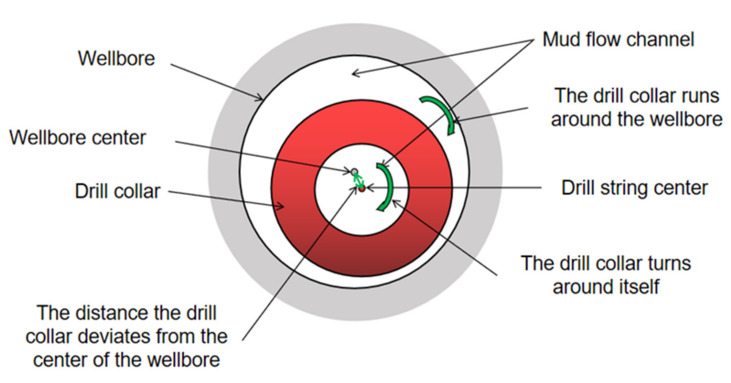
Profile of the drill collar and wellbore relative to each other.

According to Figure 1b and Figure 2, the operating states of the instrument while drilling in the wellbore mainly include sinusoidal buckling, tilt, eccentric uncenter in the wellbore, self-rotation around the center of the drill collar, rotation around the center of the wellbore, and combined rotation in several ways. The eccentric vortex of the drill string caused by friction resistance between the drill string and drill bit is also one of the main operating conditions of the downhole drill string. These complex operating attitudes change with time due to rocks breaking the drill bit and the friction of the borehole wall. Based on years of research by oil industry scientists [1,2,3,4,5,6], we have summarized the common operating types of drill string near the downhole DCWD in the Table 2.

Eight common drill string operating state combinations can be combined in the figure above. In the following sections, after the DCWD attitude is obtained using the multi-sensor layout, we will analyze each of the above scenarios for drill string misalignment.

### 2.4. Influence of Eccentric Factors on Measurement

**Figure 3:**
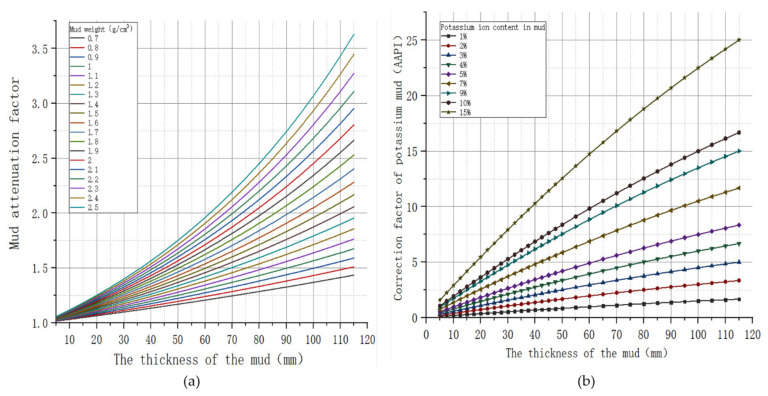
(**a**) Gamma mud gravity correction; (**b**) Gamma correction pattern of potassium-based mud.

The eccentricity of the measurement sensor has a great influence on the measurement of the instrument. For imaging while drilling gamma ray measurements, natural gamma rays from the rock formation travel through the annulus in the drill string and wellbore to photomultiplier tubes in the instrument, triggering pulses that are used to count the rays. Due to the tungsten alloy shield on the outside of the gamma photomultiplier tube, only a narrow window is allowed to enter the ray, and the media in the wall of the hole will attenuate the incoming ray. As shown in Figure 3a, the effect of mud thickness on natural gamma attenuation is exponentially influenced by the density and thickness of the mud on the gamma count attenuation. The presence of potassium ions in the potassium-based mud, as shown in Figure 3b, also affects the measurement results, increasing in direct proportion to the mud thickness and the proportion of potassium ions in the mud. Therefore, it seems that only by accurately obtaining the specific distance between the imaging measurement sensor and the borehole wall can the original data be corrected, and the real formation response results be obtained. According to the above discussion, the original theory can only qualitatively analyze the drill string from a macro perspective and cannot obtain the real-time distance from the sensor on the drill collar under study to the borehole wall.

## 3. Borehole Eccentricity Multi-Sensor Measurement Principle and Measurement Method

### 3.1. Composition of Sensors for Measuring Drill String Eccentricity

The IMWD sensor is located on the drill collar between the drill string and the power drill string. The drill collars in this part move in the wellbore with complex motions such as tilt, bend, rotation, and revolution. The traditional diameter sensor can measure the diameter near the sensor [17]. Past applications have focused on surface dimensional changes in the wellbore, with no focus on measuring the operational characteristics of the drill string. Due to the inability to obtain the eccentric and tilting characteristics of drill collars in the wellbore, the imaging sensor while drilling near the deviation diameter measuring point cannot provide real-time diameter information. In this paper, a multi-sensor layout measurement method is proposed to measure the movement characteristics of drill collars in the wellbore, and the distance between drill collars and the wellbore wall corresponding to different positions and tool face angles of drill collars in imaging while drilling logging is obtained. The imaging measurement sensor in the drill collar can then be used to obtain the eccentricity distance for environmental correction using a correction plate, similar to that shown in Figure 3a,b (gamma) for a more realistic formation response.

**Figure 4:**
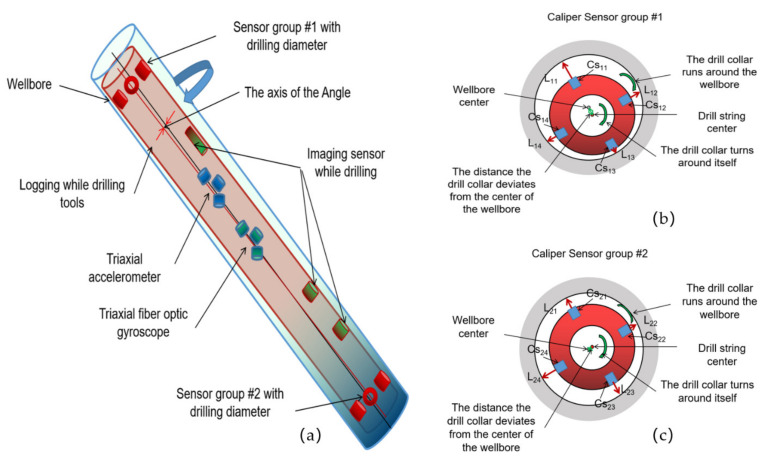
(**a**) Schematic diagram of the multi-sensor layout of the drill string eccentricity measurement system; (**b**) Diagram of upper sensor set eccentricity; (**c**) Diagram of down sensor set eccentricity.

As shown in Figure 4a, the method consists of two sets of ultrasonic diameter sensors installed at each end of the IWD drill collar to obtain the actual shape of the wellbore. It can also be used to measure the eccentric attitude and distance of drill collars in the wellbore. A fiber-optic gyro attitude sensor set was installed in the middle of the drill collar, as shown in Figure 4a, to obtain information on the inclination, azimuth, and measurement tool face at the drill collar location. Communication cables are connected between the circuits of these sensors, and the measured and calculated information is transmitted in real time on the communication bus. The measured and calculated attitude and eccentricity distance of the drill string can be acquired in real time by the imaging sensor while drilling on the side wall of the drill collar, which can be integrated to complete the eccentricity correction of the imaging sensor while drilling. As can be seen from Figure 4b,c, the center of the drill string corresponding to the drill collar cross-section of the two groups of ultrasonic sensors does not coincide with the center of the wellbore. In the following sections, we will elaborate the calculation methods of the drill collar center, the wellbore center, the angle between the drill collar axis and the wellbore axis on the two interfaces, the self-rotation of the drill collar, and the revolution of the drill collar around the wellbore center.

**Figure 5:**
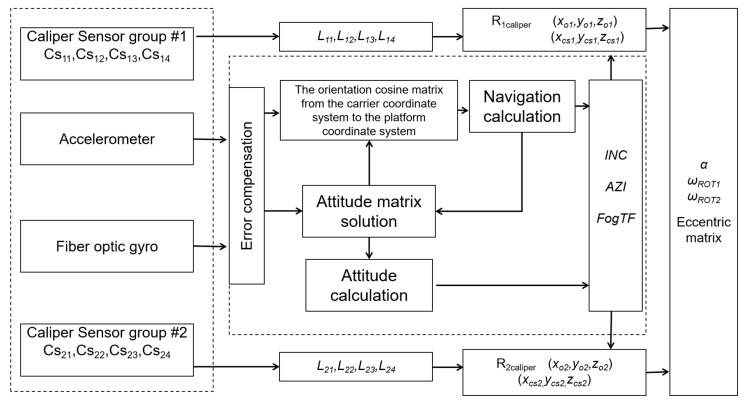
Schematic diagram of the multi-sensor layout of the drill string eccentricity measurement data processing.

Figure 4 and Figure 5 show the schematic diagrams and data processing chart of the multi-sensor layout drill string eccentricity measurement system, respectively. The attitude sensor installed in the middle of the drill collar consists of three orthogonal acceleration sensors and three orthogonal fiber-optic gyro sensors. The measured accelerometer values *G_x_, G_y_, G_z_* and angular velocity values *ω_x_, ω_y_, ω_z_* were obtained. Using these six data points, the drill string’s inclination, azimuth, and measurement tool face angle from each sensor to the wellbore can be calculated. Each set of ultrasonic sensors consists of four ultrasonic ranging sensors, each of which is uniformly installed around the wellbore at 90° intervals to obtain four measurement distances simultaneously. Combined with the well inclination, azimuth, and measurement tool surface measured by the attitude sensor in the middle, we can calculate the diameter of the wellbore, the middle coordinates of the drill collar center, and the four points in the wellbore center on the section of the two groups of ultrasonic sensors. Thus, the included angle between the wellbore pivot line segment and the drill collar center line segment can be obtained. The angular velocity of the drill collar around the center of the wellbore can be calculated by using the changes in the coordinates of the center of the wellbore measured at different times. The angular velocity of the drill string rotation can be calculated using the change in the tool face angle measured by the fiber-optic gyroscope. Through the measurement and calculation of the above parameters, the eccentric attitude matrix of the drill string in the wellbore at a specific time can be further obtained in real time. It can be seen that the multi-sensor layout measurement and measurement data processing methods discussed in this article can obtain the changing eccentricity matrix from a microscopic perspective in real time and promote the dynamic eccentricity of the drill collar from qualitative analysis to real-time monitoring and measurement calculation. Thus, we can clearly obtain the drill string in the wellbore running characteristics and eccentricity characteristics.

### 3.2. Acquisition of Multi-Directional Diameter and Drill Collar Attitude

#### 3.2.1. Obtain Borehole Inclination, Azimuth, and Tool Face

Fiber-optic gyro is a kind of optical gyroscope based on the Sagnac effect [24,25,26]. The Sagnac effect refers to the two-way transmission of two beams of light in a closed optical circuit along the clockwise and counterclockwise direction due to an optical loop around the perpendicular to the plane of the axis of rotation; the change in the phase difference, the phase difference and closed optical circuit is proportional to the rotation rate. In this paper, the quaternion method used to analyze the corresponding well inclination, azimuth, and tool face angle. We assume that the rotation quaternion of the drill collar carrier coordinates relative to the platform coordinate system is:

(2) $$Q=q_{0}+q_{1}{\vec{i}}_{b}+q_{2}{\vec{j}}_{b}+q_{3}{\vec{k}}_{b}$$

where $q_{0}^{},q_{1}^{},q_{2}^{},q_{3}^{}$ are the real part of the quaternion and ${\vec{i}}_{b},{\vec{j}}_{b},{\vec{k}}_{b}$ are the imaginary part of the quaternion.

A correction of Q can be achieved by solving the following quaternion differential equation:

(3) $$\left[\begin{array}{c} {\dot{q}}_{0}\\ {\dot{q}}_{1}\\ {\dot{q}}_{2}\\ {\dot{q}}_{3} \end{array}\right]=\frac{1}{2}\left[\begin{array}{cccc} 0 & -\omega_{x}^{b} & -\omega_{y}^{b} & -\omega_{z}^{b}\\ \omega_{x}^{b} & 0 & \omega_{z}^{b} & -\omega_{y}^{b}\\ \omega_{y}^{b} & -\omega_{z}^{b} & 0 & \omega_{x}^{b}\\ \omega_{z}^{b} & \omega_{y}^{b} & -\omega_{x}^{b} & 0 \end{array}\right]\left[\begin{array}{c} q_{0}\\ q_{1}\\ q_{2}\\ q_{3} \end{array}\right]$$

where $\omega_{x}^{b},\omega_{y}^{b},\omega_{z}^{b}$ represent the angular velocity components of the carrier coordinate system relative to the geographic coordinate system along each axis.

The initial value of the quaternion is determined by the elements of the attitude matrix determined in the initial alignment, and the corresponding elements of the quaternion and attitude matrix are equal to each other.

Equation (4) can be used to calculate $q_{0}^{},q_{1}^{},q_{2}^{},q_{3}^{}$, and the strapdown matrix ***T*** can be calculated according to the following equation:

(4) $$T=\left[\begin{array}{ccc} q_{0}^{2}+q_{1}^{2}-q_{2}^{2}-q_{3}^{2} & 2\left(q_{1}q_{2}-q_{0}q_{3}\right) & 2\left(q_{1}q_{3}+q_{0}q_{2}\right)\\ 2\left(q_{1}q_{2}+q_{0}q_{3}\right) & q_{0}^{2}-q_{1}^{2}+q_{2}^{2}-q_{3}^{2} & 2\left(q_{2}q_{3}-q_{0}q_{1}\right)\\ 2\left(q_{1}q_{3}-q_{0}q_{2}\right) & 2\left(q_{2}q_{3}+q_{0}q_{1}\right) & q_{0}^{2}-q_{1}^{2}-q_{2}^{2}+q_{3}^{2} \end{array}\right]$$

The strapdown matrix *T* is the attitude matrix *C_n_^b^*.

(5) $$C_{b}^{n}=\left[\begin{array}{ccc} q_{0}^{2}+q_{1}^{2}-q_{2}^{2}-q_{3}^{2} & 2\left(q_{1}q_{2}-q_{0}q_{3}\right) & 2\left(q_{1}q_{3}+q_{0}q_{2}\right)\\ 2\left(q_{1}q_{2}+q_{0}q_{3}\right) & q_{0}^{2}-q_{1}^{2}+q_{2}^{2}-q_{3}^{2} & 2\left(q_{2}q_{3}-q_{0}q_{1}\right)\\ 2\left(q_{1}q_{3}-q_{0}q_{2}\right) & 2\left(q_{2}q_{3}+q_{0}q_{1}\right) & q_{0}^{2}-q_{1}^{2}-q_{2}^{2}+q_{3}^{2} \end{array}\right]$$

The definition domains of the well inclination angle (*Inc*), azimuth angle (*Azi*), and tool face angle (*FogTf*) are: 0°~180°, 0°~+360°, and 0°~360°, respectively. *Inc* = *θ*, *Azi* = *Ψ*, *FogTf* = *γ*, so the attitude matrix can be *θ, Ψ, γ.*

(6) $$C_{b}^{n}=\left[\begin{array}{ccc} \cos\theta \cos\psi +\sin\gamma \sin\psi \sin\theta & \sin\psi \cos\theta & \sin\gamma \cos\psi -\cos\gamma \sin\psi \sin\theta \\ -\cos\theta \sin\psi +\sin\gamma \cos\psi \sin\theta & \cos\psi \cos\theta & -\sin\gamma \sin\psi -\cos\gamma \cos\psi \sin\theta \\ -\sin\gamma \cos\theta & \sin\theta & \cos\gamma \cos\theta \end{array}\right]$$

The setup is:

(7) $$C_{b}^{n}=\left[\begin{array}{ccc} T_{11} & T_{12} & T_{13}\\ T_{21} & T_{22} & T_{23}\\ T_{31} & T_{32} & T_{33} \end{array}\right]$$

Then:

(8) $$\left\{\begin{array}{l} Inc=\arcsin\left(T_{32}\right)\\ FogTf=\arctan\left(-\frac{T_{31}}{T_{33}}\right)\\ Azi=\arctan\left(\frac{T_{12}}{T_{22}}\right) \end{array}\right.$$

#### 3.2.2. Calipers Sensor

**Figure 6:**
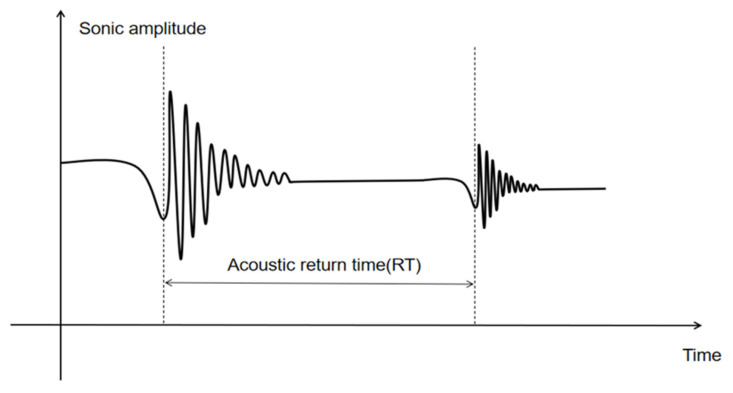
Ultrasonic caliper measurement echo.

As shown in Figure 6, the sensor used for measuring along the drilling calipers is an ultrasonic sensor [27,28,29]. The ultrasonic sensor we selected operates at 250 kHz, and when the instrument is operating the sensor sends a series of acoustic pulses to the borehole wall. This signal is reflected off the wall of the wellbore and is picked up by ultrasonic sensors. Using the time difference between the transmission speed of sound waves in the mud and the transmission and acceptance, we can calculate the distance from the sensor to the borehole wall, as shown in Equation (9).

(9) $$l_{s}=\frac{1}{2}V_{s}R_{T}$$

where *Vs* is the transmission speed of sound wave in mud, mm/s; *R_T_* is the time from the sensor sending the pulse to receiving the reflected signal from the borehole wall, ms.

The distance from the eight sensors in a set of four to the wellbore wall can be calculated by firing and receiving four sensors from the two sets of calipers sensors at the same time: *L_11_, L_12_, L_13_, L_14_, L_21_, L_22_, L_23_,* and *L_24_*. In the following sections, we will use these basic measurements to calculate the drill string operating characteristics and eccentricity.

### 3.3. Methods and Equations for Determining the Eccentricity of Two Groups of Sensors

**Figure 7:**
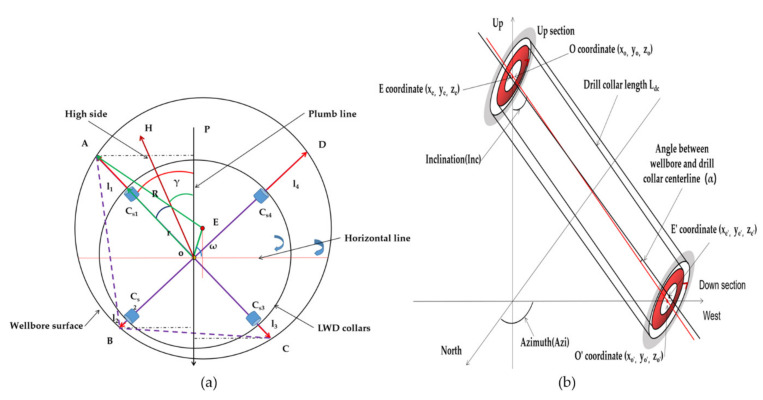
(**a**) Drill string eccentricity diagram section; (**b**) The drill string is eccentric in the wellbore.

As shown in Figure 7a, points A, B, C, and D correspond to points on the borehole wall in the direction corresponding to the ultrasonic calipers’ sensor. According to the principle of three points to determine a circumscribed circle and the four vertices, four triangles can be determined: ΔABC, ΔBCD, ΔCDA, and ΔDAB. We first select ΔABC to calculate the shaft’s center and radius. Firstly, the center of the instrument is set as point O, and the coordinate is (0, 0, 0).

In Figure 7a, point H is the high edge of the instrument in line with 0° of the tool surface corresponding to the gyro measuring instrument. Points A, B, and C are the corresponding measurement points of ultrasonic sensors *C_S1_*, *C_S2_*, and *C_S3_* on the borehole wall. Angle ∠HOA is the measurement tool face angle difference of the instrument, which can be measured on the ground and is a known fixed value. ∠POH is the tool face angle of the drilling tool, which can be measured using a gyro. The numerical value is *FogTf*, and the calculation method is shown in Equation (8). Thus, it can be concluded that:

(10) ∠POA=∠POH+∠HOA

Since the angle of each sensor is perpendicular to the center, the following A, B, and C coordinate points can be obtained:

(11) $$\left\{\begin{array}{lll} -L_{11}\sin∠\mathrm{AOP}\;,\; & L_{11}\cos∠\mathrm{AOP}\; & A_{\mathrm{point}}\\ L_{12}\sin(90-∠\mathrm{AOP})\;,\; & L_{12}\cos(90-∠\mathrm{AOP}) & B_{\mathrm{point}}\\ L_{13}\sin(180-∠\mathrm{AOP})\;,\; & L_{13}\cos(180-∠\mathrm{AOP}) & C_{\mathrm{point}} \end{array}\right.$$

where *L*_11_ = *l*_11_ + *r_dc_*; *L*_12_ = *l*_12_ + *r_dc_*; *L*_13_ = *l*_13_ + *r_dc_*. *l*_11_, *l*_12_, and *l*_13_ are the distances measured by sensors *C_S1_, C_S2,_ and C_S3_,* mm. *r_dc_* is the drilling tool radius in mm.

We set:

(12) $$\left[\begin{array}{l} x_{a}{\;y}_{a}\\ x_{b}{\;y}_{b}\\ x_{c}{\;y}_{c} \end{array}\right]=\left[\begin{array}{ll} -L_{11}\sin∠\mathrm{AOP}\; & L_{11}\cos∠\mathrm{AOP}\\ L_{12}\sin(90-∠\mathrm{AOP}) & L_{12}\cos(90-∠\mathrm{AOP})\;\\ L_{13}\sin(180-∠\mathrm{AOP}) & L_{13}\cos(180-∠\mathrm{AOP})\; \end{array}\right]$$

According to the equation for finding the center of the circumferential circle of a triangle, we can obtain:

(13) $$\left\{\begin{array}{l} y_{e}=\frac{L_{a}+L_{b}+L_{c}}{2}\\ x_{e}=\sqrt{y_{e}(y_{e}-L_{a})(y_{e}-L_{b})(y_{e}-L_{c})}\\ R=\frac{L_{a}L_{c}L_{c}}{4x_{e}}\\ \omega_{z}=arctg\frac{x_{o}}{y_{o}} \end{array}\right.$$

(14) $$\left\{\begin{array}{l} L_{a}=\sqrt{{\left(x_{a}-x_{b}\right)}^{2}+{\left(y_{a}-y_{b}\right)}^{2}}\\ L_{b}=\sqrt{{\left(x_{a}-x_{c}\right)}^{2}+{\left(y_{a}-y_{c}\right)}^{2}}\\ L_{c}=\sqrt{{\left(x_{b}-x_{c}\right)}^{2}+{\left(y_{b}-y_{c}\right)}^{2}} \end{array}\right.$$

where *X_e_* and *Y_e_* are the coordinates of the wellbore center relative to the center of the drill string in mm, *R* is the radius of the wellbore in mm, and *ω_z_* is the angle from the center of the drill collar to the center of the wellbore.

Further in space, we can get the spatial coordinates of E (*X_E_, Y_E_, Z_E_*). Similarly, we can further use four triangles to find the coordinates of four central points: *E1, E2, E3,* and *E4*. The final central coordinates can be weighted and averaged to obtain the final *X_E_, Y_E_,* and *Z_E_*. The four sensors give the four radii, which are averaged to get the final *R*.

As shown in Figure 7b, OO′ is the center line of the drill collar and O′ is the center point of the drill collar at the second calipers sensor. Using the drill string deviation and orientation obtained by Equation (9), we can obtain the coordinates of O′ (*X_O′_, Y_O′_, Z_O′_*) on the spatial coordinates.

The equation is as follows:

(15) $$\left\{\begin{array}{l} X_{o^{\prime }}=L_{\mathrm{dc}}\sin(Inc)\cos(Azi)\\ Y_{o^{\prime }}=L_{\mathrm{dc}}\sin(Inc)\sin(Azi)\\ Z_{o^{\prime }}=L_{\mathrm{dc}}\cos(Azi) \end{array}\right.$$

Similarly, by using Equations (10)–(14) we can calculate the coordinates of E′ point (X_E′_, Y_E′_, Z_E′_) and the well calipers R′ of this section. Using the above steps, we can obtain the coordinates of the drill collar center O and the wellbore center E on the section of the first group of sensors and the coordinates of the drill collar center O′ and the wellbore center E′ on the section of the second group of sensors. The included angle between the drill collar centerline and the wellbore centerline is the included angle between the space segment OO′ and EE′. Let us call the vector of OO prime $\vec{O}$, (*O*_1_, *O*_2_, *O*_3_); the EE′ vector is $\vec{E}$, (*E*_1_, *E*_2_, *E*_3_).

The included angle α between two line segments is:

(16) $$\alpha =\arccos\left(\frac{O_{1}E_{1}+O_{2}E_{2}+O_{3}E_{3}}{\sqrt{O_{1}{}^{2}+O_{2}{}^{2}+O_{3}{}^{2}}\times \sqrt{E_{1}{}^{2}+E_{2}{}^{2}+E_{3}{}^{2}}}\right)$$

### 3.4. Representation of Eccentricity Matrix at a Measuring Point Along the Drill Collar

As shown in Figure 7b, when the central coordinate O, O′ of the drill string where the upper and lower sensor section is located and the coordinate E, E′ of the wellbore and the calipers of the wellbore are calculated by Equations (10)–(16), we can calculate the tool face angle and the distance *Len* (along the axial direction of the drill collar) from the wellbore wall. Its eccentricity matrix is expressed as follows:

(17) $$\left[\begin{array}{ccc} Dep_{1} & Tf_{11} & Len_{11}\\ Dep_{2} & Tf_{12} & Len_{12}\\ Dep_{3} & Tf_{13} & Len_{13}\\ \cdot & \cdot & \cdot \\ \cdot & \cdot & \cdot \\ \cdot & \cdot & \cdot \\ Dep_{n} & Tf_{1n} & Len_{1n} \end{array}\right]\left[\begin{array}{ccc} Dep_{1} & Tf_{21} & Len_{21}\\ Dep_{2} & Tf_{22} & Len_{22}\\ Dep_{3} & Tf_{23} & Len_{23}\\ \cdot & \cdot & \cdot \\ \cdot & \cdot & \cdot \\ \cdot & \cdot & \cdot \\ Dep_{n} & Tf_{2n} & Len_{2n} \end{array}\right]\left[\begin{array}{ccc} Dep_{1} & T_{m1} & Len_{m1}\\ Dep_{2} & T_{m2} & Len_{m2}\\ Dep_{3} & Tf_{m3} & Len_{m3}\\ \cdot & \cdot & \cdot \\ \cdot & \cdot & \cdot \\ \cdot & \cdot & \cdot \\ Dep_{n} & Tf_{mn} & Len_{mn} \end{array}\right]$$

where *Dep_1_~Dep_n_* are the length of each measuring point of the drill collar where the imaging measurement sensor is located in mm and *Tf_11_~Tf_mn_* are the measurement tool face angles, ranging from 0° to 360°. *Len_11_~Len_mn_* are the distance from the drill collar to the wellbore wall corresponding to different depths and tool face angles, unit mm.

## 4. Simulation Analysis and Field Test of Drill String Eccentricity State

On the basis of the above theoretical analysis, we simulate the position of the drilling string in the wellbore where the imaging instrument is located. The experiment was carried out in three groups. In the first group, it is assumed that the drill string is not buckling, the drill collar axis is parallel to the wellbore axis, and the included angle is zero. When the drill collar rotates in the wellbore, the distance distributed along the surface of the drill collar to the wellbore is centered rotation and eccentric rotation, respectively. In the second group, it is assumed that the drill string does not buckle, the drill collar axis and the wellbore axis are not parallel, and they are, respectively, tilting rotation, tilting rotation, and revolution. The third group is the operation state of the drill collar under compression, which is the rotation of the drill string under buckling and the rotation and revolution of the drill string under buckling.

In order to verify the measurement and calculation methods mentioned in this paper, we used the imaging while drilling simulation measurement device of China University of Petroleum. The device consists of a logging-while-drilling attitude scanning device and a simulated wellbore, which can be fitted with some ultrasonic diameter sensors to scan the borehole wall to simulate the drill string’s eccentricity, tilt, rotation, and revolution in the wellbore. At the same time, in order to further simulate the running state of the drill collar in the well bore where the logging sensor is located, MATLAB and Visual Basic 2015 were used to write and simulate the eccentricity while drilling program software. The software can input the measurements of wellbore dimensions, drill string dimensions, drill string attitude, and ultrasonic well diameter mentioned in Section 3 to obtain the drillstring eccentricity distance matrix mentioned in Equation (17). The matrix establishes an eccentric set of the corresponding IMWD and LWD sensors corresponding to the wellbore wall deployed longitudinally along the drill string, with each measured depth around 360 degrees. 

In the following figures, there wre two forms to show the eccentricity distance. One form was to expand in the order of records, the abscissa was the record number, the ordinate was the periodic change of the angle, and the chromatogram shows the change of the eccentric distance. The other was that the abscissa indicates the length of the drill collar, the ordinate indicates the angle change around the drill collar, and the color spectrum indicates the distance from the corresponding point on the surface of the drill collar to the borehole wall.

The commonly used BHA is selected for the simulation setting conditions, such as BHA No.3 in Table 1. The drill bit diameter was 215.9 mm, the drill collar diameter was 172 mm, and the drill collar length was 20 m. The size of the drill bit, drill collar diameter, drill collar length, rotation speed, and revolution speed were input into the software. The simulation outputs the eccentric distance matrix of the sensor to the wellbore, distributed along the surface of the drill collar under eccentric, tilt, and buckling conditions. A total of 72,000 observation points were set down along the drill collar with an interval of 50 mm in length and an angle interval of 2° around the drill collar.

### 4.1. Analysis of Eccentricity Characteristics in Case of No Bending of Drill String and Zero Included Angle

In this experiment, the assumption is that when the drill string does not buckle at the observation point, the angle between the two axis lines is 0.

#### 4.1.1. Center Rotation

The posture condition of the simulation drill string is the center rotation of the drill string and the rotation speed is 60 RPM. The eccentricity is uniform at 21.95 mm.

**Figure 8:**
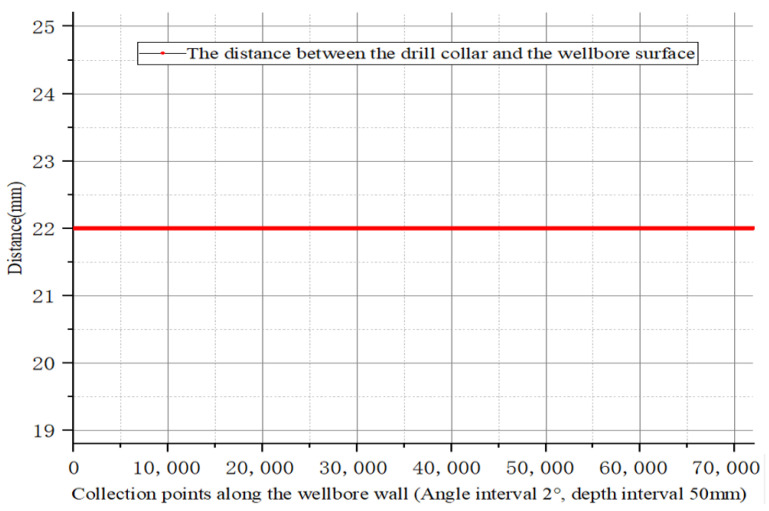
The eccentric distance of the drill collars running in the center of the wellbore.

At this point, the drill collars rotate in the center of the drill string without revolution, and the distance between the sensors distributed on the drill collars and the wellbore is uniform at 21.95 mm, as can be seen in Figure 8. This state is the ideal drill collar running state in the well and rarely exists under actual working conditions.

#### 4.1.2. Eccentric Rotation

In this simulation, the posture condition of the drill string is that the center line of the drill collar is deviated but parallel to the axis of the wellbore, the angle between the two axis lines is zero, and the rotation speed is 60 r/min. The eccentricity is 21.925 mm.

**Figure 9:**
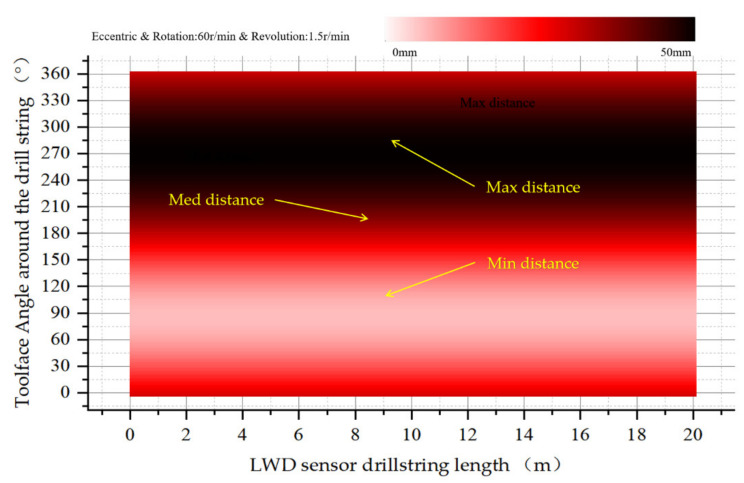
Eccentricity distance distribution along the surface of the drill collar during eccentric rotation.

The drill collar rotates eccentrically on the right side of the wellbore without revolution. The maximum tool face angle on the drill collar was measured at 270°, and the maximum distance between the drill collar and the wellbore wall was 43.9 mm. The minimal distance occurs at 90°, 0 mm. The distance between the drill collar and the borehole wall is related to the tool face and independent of the drill collar length, as can be seen in Figure 9.

**Figure 10:**
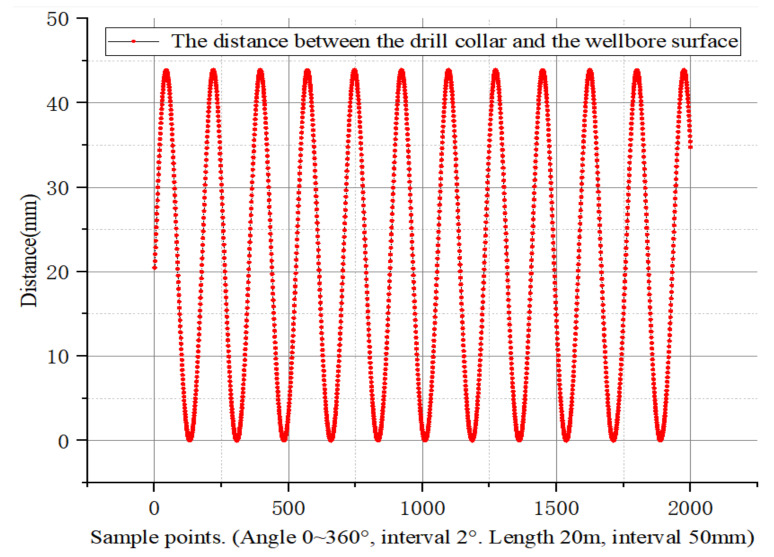
Eccentricity distance in order of recorded data.

The simulation data are arranged and expanded in order. The angle interval is 2° and the depth interval is 50 mm. The first 2000 data obtained are shown in the following Figure 10.

**Figure 11:**
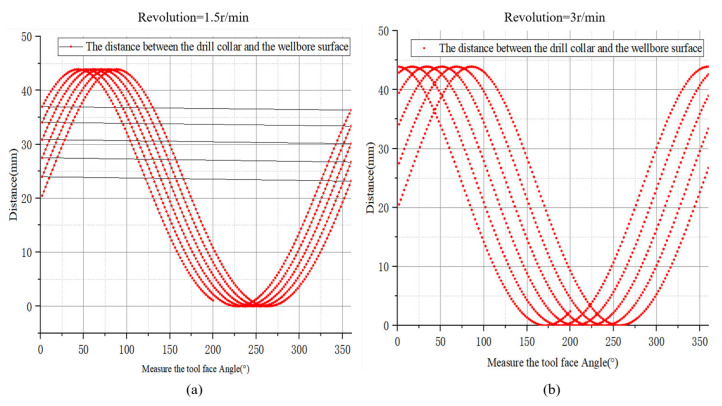
(**a**) Display the eccentricity in the order of recording according to 1.5 RPM revolution; (**b**) Display the eccentricity in the order of recording according to 3 RPM revolution.

Figure 11a shows the phase displacement of the tool face with the drill collar eccentricity at 1.5 r/min revolution. Figure 11b shows the phase displacement change of the tool face with the required eccentricity at 3 r/min revolution in the drill collar eccentricity state. It can be seen that under the eccentric condition, the phase displacement increases with the revolution speed in the case of additional revolution.

### 4.2. Analysis of Eccentricity Characteristics in the Case of No Bending of Drill String and Included Angle Greater Than Zero

There is no bend in the drill string, but there is an angle between the collar axis and the wellbore axis.

#### 4.2.1. Tilting Rotation

**Figure 12:**
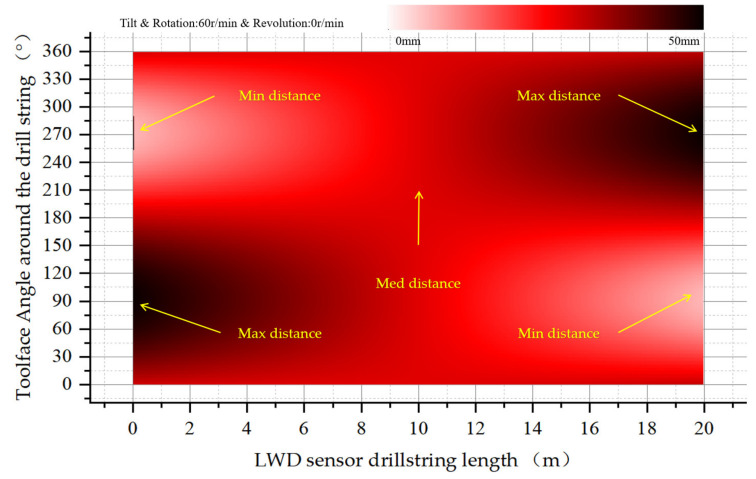
Eccentricity distribution of drill collars as they tilt in the wellbore.

The drill string tilts and rotates in the wellbore, and there is no revolution around the wellbore, with a rotation rate of 60 RPM. The upper part of the drill collar deviates to the left and the lower part deviates to the right. The maximum starting distance is 90 degrees from the tool face and the minimum distance is 270 degrees from the tool face. The change begins along the drill collar axis, centered in the middle. In the lower part of the drill collar to the right, the maximum eccentricity occurs at the 270-degree tool face and the minimum eccentricity occurs at the 90 degree tool face. The tool faces of the drill collars are sinusoidal at the upper and lower ends and rotate in the middle. The collar-to-wellbore eccentricity distribution is shown in Figure 12.

#### 4.2.2. Tilting Rotation 60 RPM and Revolution

**Figure 13:**
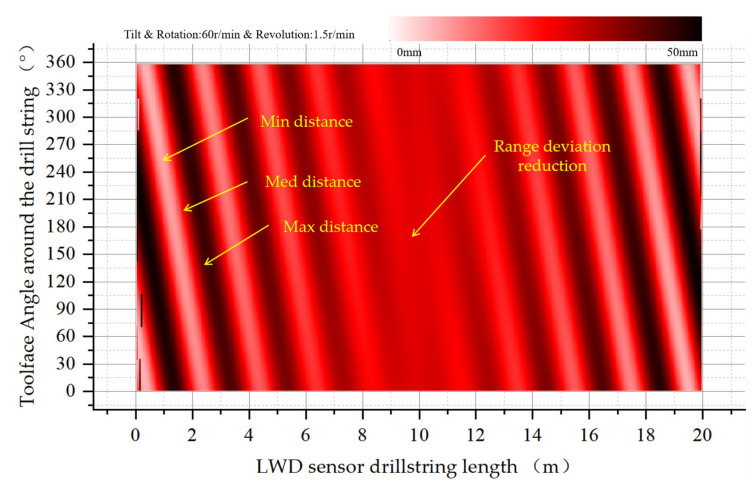
Eccentricity distribution of drill collars in the wellbore. with oblique rotation and additional revolution at a speed of 1.5 RPM.

The drill collars were tilted in the wellbore with a rotation rate of 60 RPM and the initial conditions were the same as in Section 4.2.1. On this basis, the drill collar axis around the wellbore axis occurred at 1.5 RPM revolution. As shown in Figure 13, along the axial direction of the drill string the oblique rotation and revolution appear to be coupled and undergo periodic changes. Along the axial direction of the drill string, the eccentricity changes sharply at both ends of the drill string but less in the middle.

**Figure 14:**
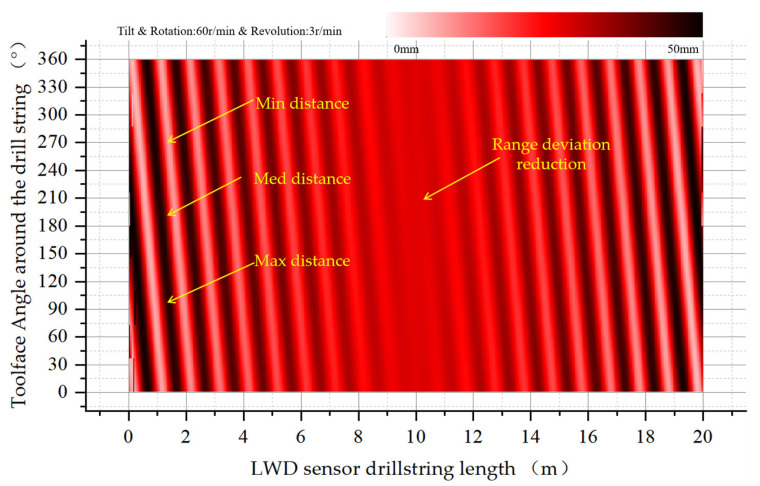
Eccentricity distribution of drill collars in the wellbore, with oblique rotation and additional revolution at a speed of 3 RPM.

When increasing the revolution speed to 3 RPM, as shown in Figure 14. It can be seen that the frequency of periodic changes at both ends of the drill collar along the axial direction of the drill collar increases.

### 4.3. Analysis of Eccentric Characteristics of the Drill String with Bending

The test drill string buckled under stress. The bus of the upper and lower drill collars is on the axis line of the wellbore, and the middle buckling of the drill collars touches the wellbore wall on the right side of the wellbore. The drill collar rotation rate is 60 RPM.

#### 4.3.1. Buckling Rotation without Revolution

**Figure 15:**
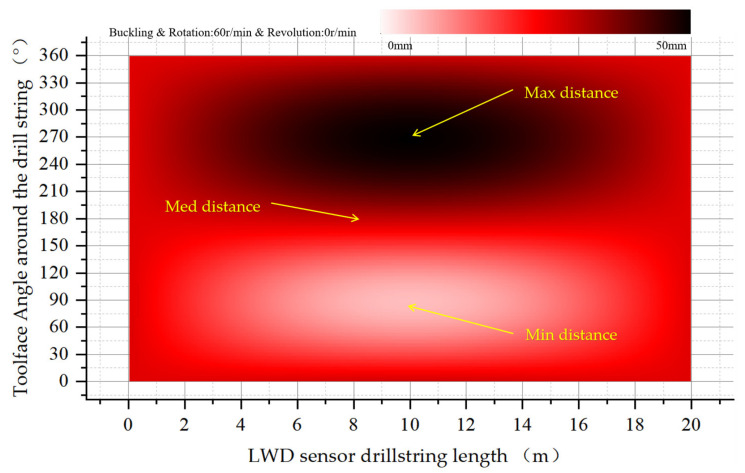
Eccentricity distribution of drill collars during buckling rotation (no revolution) in wellbore.

As shown in Figure 15, the distance between the two ends rotates along the centerline of the wellbore with little change in eccentricity. Along the axis direction to the middle of the drill collar, the eccentric deviation gradually presents sinusoidal variation, and the amplitude is the largest in the middle of the drill collar.

#### 4.3.2. There Is Revolution in Buckling

**Figure 16:**
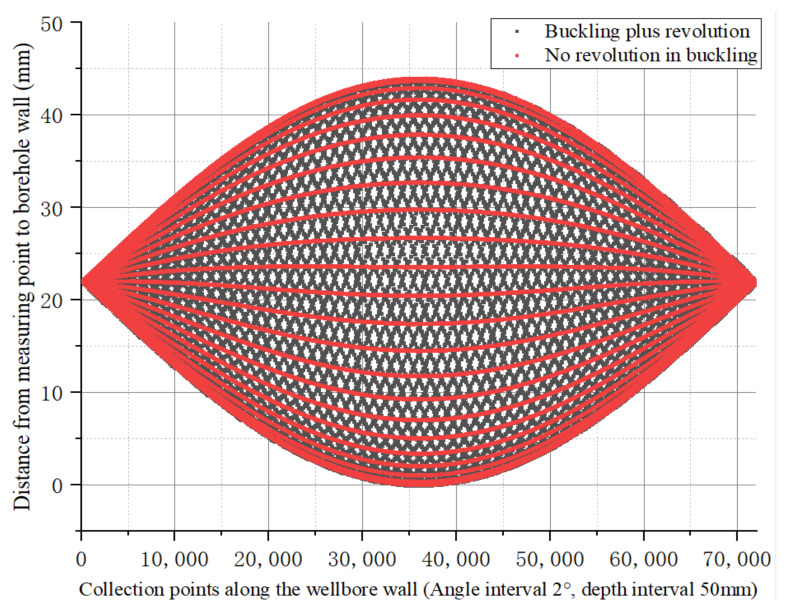
Comparison of the eccentricity distribution of drill collars recorded in the order of buckling rotation and additional revolution in the wellbore.

In Section 4.3.1, a revolution around the wellbore axis was added to the buckling rotation of the drill collar. Under such conditions, the eccentricity distribution of drill collars in the wellbore during buckling rotation (non-revolution) and additional revolution was recorded in the order shown in Figure 16. The two have the same general trend, with both ends rotating in the center of the wellbore and with the same eccentricity. The more the drill collar goes to the middle of the drill collar, the greater the eccentric vibration amplitude is, and the middle part of the drill collar keeps rotating on the borehole wall. The eccentricity drawn according to the sequence of simulation calculation and recording is similar to the variation in shuttle type, as shown in Figure 16.

**Figure 17:**
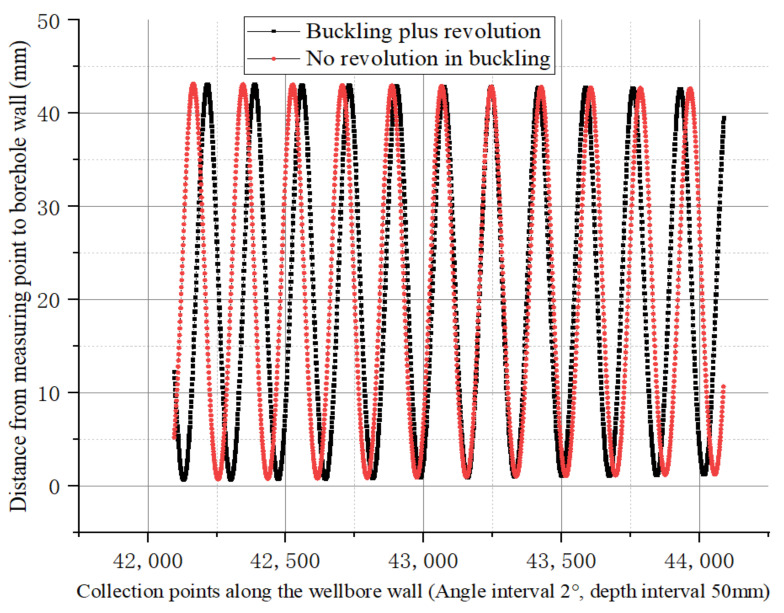
The phase change after additional revolution compared with that without additional revolution.

As shown in Figure 17, the eccentricity distances after buckling without revolution and additional revolution are compared in the order recorded, and phase changes occur between the two.

**Figure 18:**
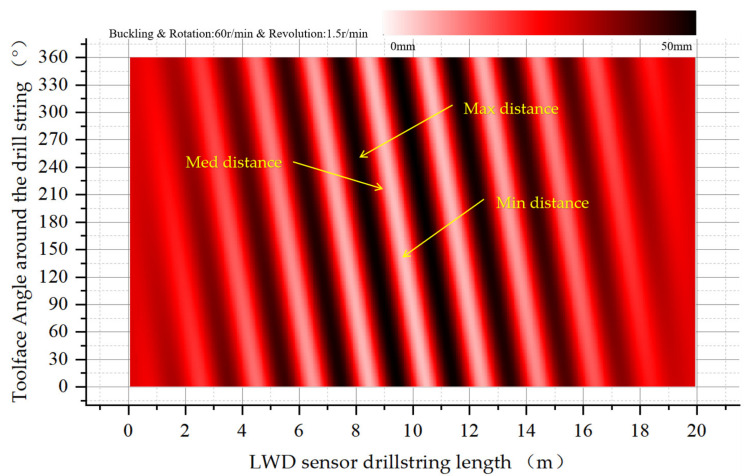
Eccentricity distribution along the drill collar surface after drill collar buckling with a revolution of 1.5 RPM.

As shown in Figure 18, under the condition of buckling a revolution around the center line of the wellbore is added to the drill string. From the two ends of the drill collar to the middle of the drill collar, the eccentricity distribution presents stripe rotation interference, with periodic changes in the maximum and minimum eccentricity.

### 4.4. Analysis and Discussion of Drill String Eccentricity Characteristics

From the simulation and test above, it can be seen that the eccentric, tilting, buckling, and other spatial postures of the drill collar in the wellbore have a great influence on the distance between the sensor on the surface of the drill collar and the wellbore wall. These spatial attitudes determine the trend and structure of the distance from the drill collar axial surface to the wellbore wall. The rotation of the drill string has no effect on the structural change in eccentricity, but it will affect the measurement of the specific sensor. Therefore, it is necessary to improve the acquisition speed to improve the resolution of the tool face under the condition of high rotation speed. The revolution of the drill string around the central axis of the wellbore is coupled with the rotation of the drill string, and the increase in revolution speed will lead to an increase in the frequency of periodic changes along the drill string axis. The above analysis was statistically compared, and the maximum eccentric distance, minimum eccentric distance, axial line crossing (uniform change around the tool face), axial change correlation along the drill collar, and periodic change of coupling were statistically analyzed according to the eight cases shown in the Table 3.

### 4.5. Field Test

**Figure 19:**
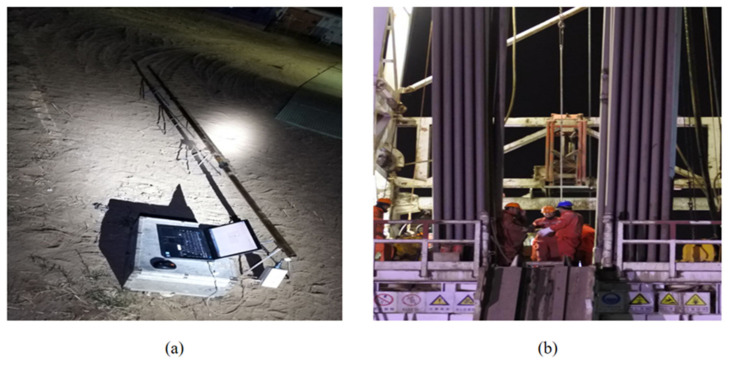
(**a**) Ground north-finding for fiber-optic gyro (FOG) while drilling; (**b**) Go down well with LWD tools.

In order to verify the results of the method mentioned in this paper in actual drilling conditions, we selected a conventional directional well in Shengli Oilfield, China, for actual testing. In the Cheng well, the fiber-optic gyro (FOG) was first assembled on the ground and the surface north-searching was performed. The tool face angle difference between the FOG and the drill collar calipers measuring machine was measured and recorded, as shown in Figure 19. After assembly, the prototype was run to the bottom of the well for measurement.

**Figure 20:**
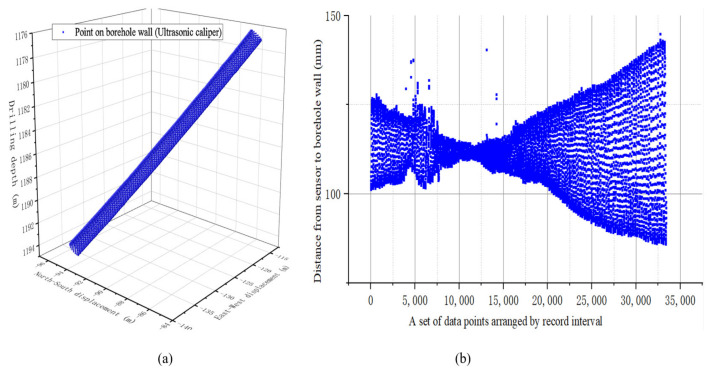
(**a**) The original caliper data of the fused gyro attitude extracted from memory; (**b**) Eccentric data set.

The downhole instruments were wired to each other and a real-time clock was used to couple the data combinations between the different sensors. The drill bit size was 216 mm, the drill collar caliper size was 172 mm, and the drill collar plus instrument sub length was 17 m. The acquisition frequency of FOG in the well was 1 kHz. The test end of the well had a depth of 1167–1225 m, a well inclination of 26.18°, and an azimuth of 32.15°. The rotation speed of the ground drill plate was 55 RPM. The attitude information and well caliper information of the test are stored in the memory. After the instrument was taken out of the ground, the data were downloaded to a computer for processing. Figure 20b shows the extracted three-dimensional caliper shape, and Figure 20b shows the eccentricity data set obtained from the memory records.

**Figure 21:**
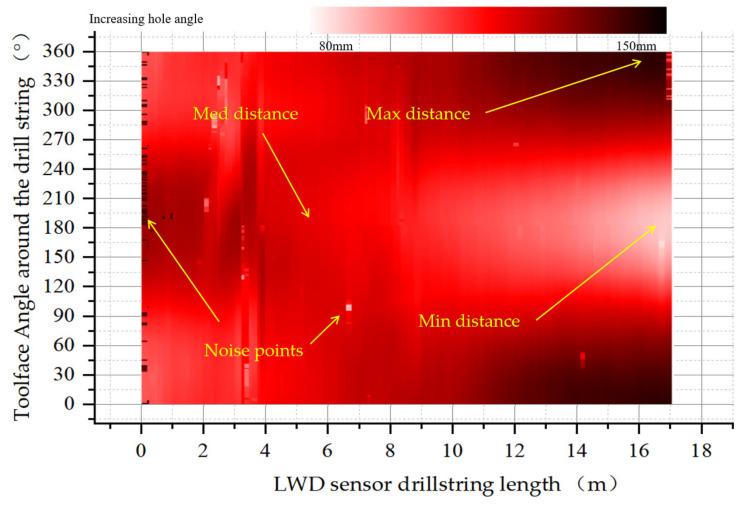
Eccentricity distribution of each tool surface along the drill collar axis.

As shown in Figure 21, the eccentricity data along the borehole axis were expanded on the 0–360° measurement tool face. It can be seen from the figure that the drill string tilted eccentrically in the wellbore at this time, and no obvious revolution phenomenon is shown. The top of the drill collars deviated from the shaft to the upper wellbore wall and intersected the pivot line of the drill collars, with the center line of the wellbore at about 5 m. As the drill string moves down, the drill collars drift toward the lower wellbore. At the bottom end of the drill collar, the drill collar’s 180° tool face is closest to the wellbore wall. At 0° and 360°, the drill collar is furthest from the wellbore wall. It can be seen that the multi-sensor combination measurement scheme proposed in this paper can be used to measure the running state of the drill string and obtain the eccentricity matrix of the drill collar distribution between the sensor groups.

## 5. Discussion and Conclusions

### 5.1. Discussion

Through the above theoretical analysis and simulation experiments, it was proven that the downhole uncentrality of drill collars in LWD leads to variation in the distance between each measuring tool face and wellbore along the axial direction of the drill collars and around the drill string. The revolution of the drill collar around the wellbore axis is coupled with the rotation of the instrument, and the period of change along the wellbore axis becomes shorter as the revolution speed increases. The multi-sensor layout eccentricity measurement method proposed in this paper can be used to analyze the running characteristics of the drill collars where the logging tool is located in real time. The rotation velocity and revolution velocity can be separated and the distance matrix between each measuring tool face and borehole wall distributed along the axis of the drill collars at each measuring point can be obtained. According to the slow elastic deformation of drill collars, the eccentricity measured in the eccentric wellbore above the drill collars and the operating state of the drill string above the IMWD can be deduced for the extension part not far from the upper and lower ends. In this paper, through continuous carrier attitude measurement of two sets of ultrasonic caliper sensors and fiber optic gyroscopes, the drill string eccentricity matrix under complex force conditions can be measured and calculated in real time. This method can greatly improve the effect of imaging sensor eccentricity correction.

### 5.2. Conclusions

Through the analysis of the eccentric characteristics of drill collars in different motion types in the well, we can draw the following conclusions:

(1) The multi-sensor layout scheme proposed in this paper can separate the rotation and revolution velocities of drill collars in the wellbore and analyze the eccentric characteristics of drill collars in the wellbore.
(2) The inclination, parallel eccentricity, buckling, and revolution of the drill string are all related to the distance between the measuring point and the borehole wall.
(3) Under the conditions of a relatively regular wellbore and a relatively regular downhole movement of drill collars, the matrix of distance between each measuring tool face and wellbore wall distributed along the axis of drill collars at each measurement point was obtained.

The multi-sensor combined drill string eccentricity measurement method described in this paper can collect and analyze the downhole operation characteristics of a drill string in real time and obtain the specific distance under the conditions of the simple movement combination of drill collars in a borehole. However, the applicability of the measurement for high-frequency lateral vibration and complex borehole wall contact relationship needs further study.

## Figures and Tables

**Table 1:** Commonly used bottom hole assembly (BHA) table for measurement while drilling and orientation.

No.	Bit Size (mm)	Downhole Motor (DM) Type (mm)	Downhole Motor (DM) Angle (°)	Drill Collar of the Instrument While Drilling (DCWD)(mm)	DCWD Length (m)	Magnification of the Hole (MH),	Range of Distance from the Sensor to the Wellbore Wall (RDSW) (mm)	Buckling
1	ϕ149.2	ϕ120	1,1.25,1.5,1.75	ϕ120	10~30	0.05~0.15	0~29.2 ± 15	Easy
2	ϕ152.4	ϕ120	1,1.25,1.5,1.75	ϕ120	10~30	0.05~0.15	0~32.4 ± 15	Easy
3	ϕ215.9	ϕ172	1,1.25,1.5,1.75	ϕ172	10~30	0.05~0.15	0~43.9 ± 20	Medium
4	ϕ241.3	ϕ203.2	1,1.25,1.5,1.75	ϕ203.2	10~30	0.05~0.15	0~38.1 ± 24	Difficult
5	ϕ311.1	ϕ203.2	1,1.25,1.5,1.75	ϕ203.2	10~30	0.05~0.15	107.9 ± 31	Difficult
6	ϕ346.1	ϕ203.2	1,1.25,1.5,1.75	ϕ203.2	10~30	0.05~0.15	142.9 ± 34	Difficult

**Table 2:** Common movement characteristics and the eccentric displacement of the drill collar (IMWDDC) operation type.

No.	Drill String Eccentric Type	Whether to Rotate Around Itself (30 r/m~75 r/m)	Additional Rotation Around the WellBore
1	Centering	Yes	Yes/No
2	Eccentricity	Yes	Yes/No
3	Tilt	Yes	Yes/No
4	Buckling	Yes	Yes/No

**Table 3:** Characteristic statistics of drill collars with different postures and additional revolutions in the wellbore.

No.		Center	Center and Revolution	Eccentric Tool Face (Tf)	Eccentric = and Revolution	Tilt	Tilt and Revolution	Buckling	Buckling and Revolution
1	Max distance	Same	Same	Specific Tf	Change	Both ends of DC	Both ends of DC	Central DC	Central DC
2	Min distance	Same	Same	Specific Tf	Change	Both ends of DC	Both ends of DC	Central DC	Central DC
3	Med distance	Same	Same	Specific Tf	Change	Central DC	Central DC	Both ends of DC	Both ends of DC
4	Drill collar length correlation	No	No	No	NO	Yes	Yes	Yes	Yes
5	Change over time	No	No	No	Yes	No	Yes	No	Yes

## Data Availability

The study did not report any data.
